# Emotions are associated with the genesis of visually induced motion sickness in virtual reality

**DOI:** 10.1007/s00221-022-06454-z

**Published:** 2022-09-06

**Authors:** Mara Kaufeld, Julia Bourdeinik, Lisa Marie Prinz, Martin Mundt, Heiko Hecht

**Affiliations:** 1 Human Systems Engineering (MMS), Fraunhofer Institute for Communication, Information Processing and Ergonomics (FKIE), Zanderstr. 5, 53111 Bonn, Germany; 2 Hochschule Bonn-Rhein-Sieg, Rheinbach, Germany; 3 Psychologisches Institut, Johannes Gutenberg-Universität Mainz, Mainz, Germany

**Keywords:** Visually induced motion sickness, Virtual reality, Simulator sickness, Positive emotions

## Abstract

Visually induced motion sickness (VIMS) is a well-known side effect of virtual reality (VR) immersion, with symptoms including nausea, disorientation, and oculomotor discomfort. Previous studies have shown that pleasant music, odor, and taste can mitigate VIMS symptomatology, but the mechanism by which this occurs remains unclear. We predicted that positive emotions influence the VIMS-reducing effects. To investigate this, we conducted an experimental study with 68 subjects divided into two groups. The groups were exposed to either positive or neutral emotions before and during the VIMS-provoking stimulus. Otherwise, they performed exactly the same task of estimating the time-to-contact while confronted with a VIMS-provoking moving starfield stimulation. Emotions were induced by means of pre-tested videos and with International Affective Picture System (IAPS) images embedded in the starfield simulation. We monitored emotion induction before, during, and after the simulation, using the Self-Assessment Manikin (SAM) valence and arousal scales. VIMS was assessed before and after exposure using the Simulator Sickness Questionnaire (SSQ) and during simulation using the Fast Motion Sickness Scale (FMS) and FMS-D for dizziness symptoms. VIMS symptomatology did not differ between groups, but valence and arousal were correlated with perceived VIMS symptoms. For instance, reported positive valence prior to VR exposure was found to be related to milder VIMS symptoms and, conversely, experienced symptoms during simulation were negatively related to subjects’ valence. This study sheds light on the complex and potentially bidirectional relationship of VIMS and emotions and provides starting points for further research on the use of positive emotions to prevent VIMS.

**Supplementary Information:**

The online version contains supplementary material available at 10.1007/s00221-022-06454-z.

## Introduction

When unpleasant symptoms such as nausea, disorientation, or oculomotor discomfort occur while immersed in a virtual environment, it is referred to as cybersickness or visually induced motion sickness (VIMS) (Cha et al. 2021; Kennedy et al. 1993; Keshavarz et al. 2014). With the increasing popularity of simulation technologies, especially in virtual reality (VR) applications, VIMS poses a problem for rising numbers of viewers (Caserman et al. 2021). According to the sensory conflict theory of motion sickness, symptoms arise when sensory inputs from visual, vestibular, and proprioceptive systems are inconsistent or when they disagree with past sensory experiences (Reason 1978; Reason and Brand 1975). In VR head-mounted displays (HMDs), a visual-vestibular conflict occurs when visual information indicates self-motion while the vestibular information signals different movement or no movement at all. An alternative theory holds that VIMS is caused by persistent postural instability in controlling the body or body parts, thus implying postural instability as a necessary and sufficient condition for VIMS (Riccio and Stoffregen 1991). Adaptation and antiemetic medication have been shown to be the most successful countermeasures (Golding and Gresty 2015), but both come at a price. Antiemetics like antihistamines and anticholinergics usually trigger severe side effects such as dizziness and fatigue (Koch et al. 2018; Shupak and Gordon 2006) and are not recommended in occupational contexts or when operating machinery. Adaptation to nauseating stimuli (Heutink et al. 2019; Jannu 2015; Young et al. 2003) is time-consuming and sometimes of limited success.

Some studies have shown that pleasant stimuli like music (Keshavarz and Hecht 2014; Peck et al. 2020), odor (Keshavarz et al. 2015), and taste (Kaufeld et al. 2022a) can mitigate adverse VIMS symptoms. However, the mechanism behind these beneficial effects remains unclear. To optimize countermeasures, we need to better understand the underlying mechanisms. In the current work, we explore whether emotional modulation could be a candidate for such a VIMS-reducing mechanism. Music and odor may serve as distractors, merely diverting attention from the negative symptoms, or they could directly modify emotional states. If the latter is the case, and positive emotions are instrumental in coping with VIMS, this could be used as a starting point to develop further countermeasures and make adaption more efficient. In the following, we provide an overview of emotions and how to induce them, elaborate on evidence for relationships between emotions and VIMS, and describe the aims of the present study.

### Emotions

Emotions describe an affective state that elicits physiological adaptations, triggers cognitive processes, and often leads to goal-directed behavior (Kleinginna and Kleinginna, 1981). Both categorical and dimensional approaches provide a basis for explaining the nature of emotions. Categorical approaches divide emotions into groups or classes of distinct emotions (e.g., Cowen and Keltner 2017; Ekman 1972; Panksepp 1998). Very prominent here are the six basic emotions (anger, surprise, disgust, happiness, fear, and sadness) identified by Ekman, which are characterized by distinctive mimic expressions and are more or less culture indifferent (see e.g., Ekman 1992). The dimensional approach, in contrast, conceptualizes emotions in a few underlying dimensions. In the so-called circumplex model, emotions are distributed based on the dimensions of valence (vertical axis) and arousal (horizontal axis) in a circular model (Russell 1980). These two dimensions seem to be relatively unrelated and thus cover different aspects of the emotional experience (Barrett and Russell 1998, 1999; Lang et al. 1993; Russell 1980). Valence, on the one hand, describes the evaluation of the pleasantness of an object or an experience (Russell 1980). Arousal, on the other hand, refers to the perception of physiological activation levels during an affective experience (Barrett 1996; Feldmann 1995). Accordingly, high affective arousal can be understood as an activation of the autonomic nervous system that affects physiological state, decision making, and readiness to act (Russell 1980). In the context of the present study, positive emotions refer to affective states with positive valence and moderate arousal.

Commonly, emotions are experimentally induced by visual stimuli, music, autobiographical recall, situational procedures, or imagery (Siedlecka and Denson 2019). According to the results of a qualitative review (Siedlecka and Denson 2019) and a meta-analysis (Joseph et al. 2020), visual stimuli create the largest effect sizes. Siedlecka and Denson (2019) showed this separately for happy emotions. For instance, standardized pictures from the International Affective Picture System (IAPS) (Lang et al. 1997; Lang and Bradley 2007), pictures of facial expressions (Ekman 1992), or pre-tested videos and film sequences (Gabert-Quillen et al. 2015) offer common methods in this context. The meta-analysis by Joseph et al. (2020) highlights challenges in inducing emotions, such as the low effect sizes in inducing positive compared to negative emotions and uncertainties about how long the induced emotions are experienced.

### Emotions and VIMS

Previous findings on the possible involvement of positive emotions and VIMS primarily comprise the studies briefly mentioned above. For instance, pleasant music was shown to reduce VIMS in a study by Keshavarz and Hecht (2014). They found that music liked by the subjects, regardless of the type of music (relaxing, neutral, or stressful), significantly reduced VIMS, as compared to playing no music or music that was disliked. In addition, the results showed a trend for relaxing music to reduce VIMS. In this study, however, the emotional valence and arousal of the musical stimuli were not taken into account. When Peck et al. (2020) did so in a follow-up study, they could not find any effects of music valence and arousal on VIMS, but VIMS was significantly reduced when music liking was at its maximum, i.e., in subjects who listened to their favorite music. Note that in Peck et al.'s (2020) study, the musical stimuli were pre-tested in terms of valence and arousal, and thus it is uncertain whether the subjects felt the emotions conveyed by the music during VIMS exposure.

Keshavarz et al. (2015), who investigated olfactory stimuli (pleasant rose scent, unpleasant leather scent, or no scent) found that in subjects who perceived the odor, the pleasant scent of roses significantly reduced the severity of VIMS. These results contrast with those of Paillard et al. (2014), who tested 18 subjects in three groups with different odors (pleasant: lime, unpleasant: petrol, neutral: distilled water). Keshavarz et al. (2015) argued that the off-vertical axis rotation used by Paillard et al. (2014) to induce motion sickness could have been too strong to be mitigated by subtle countermeasures such as odors. Moreover, different odors were chosen, which might have led to the different results. The results of a more recent study support the assumption that pleasant odors can reduce VIMS (Ranasinghe et al. 2020). They used pleasant peppermint aroma in a roller coaster simulation presented via HMD. Additionally, a study by Kaufeld et al. (2022a) investigated whether chewing peppermint or ginger gum had a positive effect on VIMS. Both gums were similarly effective in alleviating VIMS, but individual ratings of gum taste were negatively related to the severity of reported VIMS symptoms. This again points to the involvement of emotional state. Taste perception is processed via the limbic system and the hypothalamus, areas which are associated with emotions (Yamamoto 2008). All of the above studies have in common that the positive effects could be attributed to the underlying emotional state, however, emotions during the provocative stimulus have not been measured directly.

Few VIMS studies have included the role of state emotions. For example, from a set of clinical predictors, Kim et al. (2021) found that expression of affect is associated with cybersickness among highly stressed people. Reuten et al. (2021) found an association between VIMS and unpleasantness on a visual analog scale during a provocative session. Even the affective appraisal of VR environments is altered by VIMS, in the sense that the environment is evaluated as rather unpleasant by affected individuals (Van der Spek et al. 2007). Furthermore, evidence suggests that individuals with higher trait or state anxiety tend to develop more severe symptoms of VIMS (Bouchard et al. 2007; Paillard et al. 2013; Passamonti et al. 2018). Evidence for the association of emotions and nausea was also found in chemotherapy patients, in whom negative emotions and poor emotion regulation were associated with greater nausea (Ashkhaneh et al. 2015; Olver et al. 2014). Moreover, people who experience high levels of positive emotions tend to experience less pain associated with chronic health conditions (Gil et al. 2004) and are more successful in coping with illness (Cohen and Pressman 2006). According to the so-called *undoing effect*, experiencing positive emotions accelerates the body’s recovery from physiological stress (Fredrickson and Levenson 1998). However, the undoing hypothesis cannot be considered a widely accepted theory (Cavanagh and Larkin 2018). Since contradictory research results were found, the undoing effect may apply only under certain so far unknown conditions.

Besides the role of valence, arousal plays an essential role in both VIMS and emotions, as both are accompanied by a sympathetic nervous system response (Barrett 1996; Bruck and Watters 2011). Increased sympathetic tone is thought to be related to the development of more severe VIMS, although the underlying mechanisms are not yet known (Mittelstaedt 2020). Bruck and Watters (2011) proposed that an increase in arousal favors a change in respiratory rate, which in turn leads to a decrease in carbon dioxide in cerebral blood flow. This reduction leads to drowsiness, which manifests itself in classic VIMS symptoms such as dizziness, fatigue, or difficulties to concentrate. Besides subjective assessment of symptoms by questionnaires, some studies attempt to examine VIMS in an objective manner by physiological measures. In particular, galvanic skin conductance as a measure of arousal is related to VIMS (Caserman et al. 2021; Gavgani et al. 2017; Kim et al. 2005; Wan et al. 2003) and is also used to capture the emotional physiological response (Egger et al. 2019).

### Aim of the present study

The present study aimed to explore in detail how an induced emotion may modulate VIMS. We chose the role of positive emotions in VIMS as psychological factor to better understand the processes behind the emergence and the management of VIMS and to ultimately derive effective behavioral countermeasures. To do so, we exposed subjects to a 14-min starfield simulation in VR. In the experimental group, we induced positive emotions with videos played beforehand and images embedded in the starfield simulation. The control group saw emotionally neutral videos and images. We collected ratings of VIMS as well as emotional valence and arousal before, during, and after simulation. We predicted that subjects experience less severe VIMS symptoms in the presence of positive emotions as compared to neutral emotions. Additionally, we hypothesized that a negative relationship between experienced positive emotions and VIMS symptoms should be present independently of the manipulation. We chose to examine the role of emotions independently of the manipulation in case the manipulation would not be successful due to the many challenges in emotion induction. To further investigate what type of emotions are associated with VIMS, we additionally surveyed and exploratively examined discrete emotions.

## Method

### Study design

Prior to the actual data collection between August and September 2021, we pre-registered the research protocol on 06/08/2021 on the website https://aspredicted.org/ with the title *Positive emotions to reduce visually induced motion sickness in virtual reality?* with the number #72241. In addition, the research protocol was approved by the institutional ethics committee of the *Fraunhofer-Gesellschaft zur Förderung der angewandten Forschung e. V., Munich* and was conducted in accordance with the Declaration of Helsinki. Before participation, all subjects gave informed consent to participate in a VR starfield study investigating prevention of VIMS, without being told the type of prevention until the very end of the experiment. They were also told that participation was voluntary and that they could discontinue the study at any time without giving a reason and without consequences. Students of the Business Psychology program at the Bonn-Rhein-Sieg University of Applied Sciences received partial course credit as compensation.

To experimentally test the relationship between VIMS and positive emotions, we chose a single-factor between-subjects design. For emotion induction, subjects were randomly assigned to one of two groups (positive or neutral). To further investigate the interaction between time and group, we also included *time* (pre–post) or the whole *time course* as a within-subjects factor. To induce the desired emotional state, we used two emotional inductions, one before and one during the 14-min starfield simulation. Standardized questionnaires and a physiological measurement method were used to measure VIMS as a dependent variable and emotions as an independent variable.

### Measures

#### VIMS measures

Questionnaires were filled in on a tablet with the help of a survey tool’s (LimeSurvey) offline version. First, the *Visually Induced Motion Sickness Susceptibility Questionnaire* (VIMSSQ) (Keshavarz et al. 2021) was administered to check for potential differences in susceptibility between the groups. The short form of the questionnaire used in our study contains five symptom items and one additional item to capture avoidance of devices. The symptom items survey the general susceptibility of experiencing nausea, dizziness, fatigue, headache, or eyestrain in adulthood when using visual displays rated on four-point Likert scales with the choice to select *never* (0), *rarely* (1), *sometimes* (2), or *often* (3). The last item asks for the frequency of active avoidance of visual displays when experiencing the listed symptoms in the past. The total score (0–18) is calculated by summing all items, with higher scale scores indicating greater vulnerability (Golding et al. 2021).

We measured VIMS using the SSQ (Kennedy et al. 1993) before (pre-SSQ) and after (post-SSQ) exposure. Collection of SSQ values prior to exposure was used to detect possible baseline group differences and to check whether subjects exhibited symptoms typical of VIMS already at the beginning of the experiment.

Additionally, we used the *Fast Motion Sickness Scale* (FMS) (Keshavarz and Hecht 2011) as a single-item scale to continuously measure VIMS during the VR simulation. The verbal rating scale was asked 15 times per subject to reflect the time course of VIMS. Subjects were instructed to focus on general discomfort, nausea, and stomach discomfort and to ignore other feelings such as excitement, fatigue, boredom, and nervousness. In addition to nausea, dizziness also represents a predominant symptom of VIMS (see e.g., Saredakis et al. 2020). For this reason, the single-item FMS-D scale (Kaufeld et al., [Bibr CR38][Bibr CR39]) was also used and queried 15 times during the simulation. Subjects were instructed to focus on dizziness and vertigo. Accordingly, subjects first indicated their current feeling of nausea and then their current feeling of dizziness. Both scales measured the degree of symptoms and ranged from 0 (*no sickness at all/ not dizzy at all*) to 20 (*frank sickness/ extreme dizziness*).

#### Emotion measures

In our study, we used the *Self-Assessment Manikin* (SAM) (Bradley and Lang 1994) and a self-developed questionnaire about discrete emotional states at different time points. The multiple measurements served as a manipulation control and monitored the emotional state over the course of the experiment. The SAM is a nonverbal picture-based questionnaire designed to measure emotional responses. We used two one-item scales that measured valence (from negative to positive) and arousal referred to the perceived intensity of sympathetic activation (from low to high). Both scales ranged from 1 (lower end of the scale) to 9 (upper end of the scale) and consisted of five different pictograms and four additional intermediate answer options. Subjects should intuitively state their current emotions. The questionnaire provides a simple method for rapid assessment of affective responses (Bradley and Lang 1994) and a Cronbach’s alpha of 0.83 (Nabizadeh Chianeh et al. 2012).

In addition, we measured the perceived intensity of discrete emotions from *not at all* (1) to *extremely* (5). At the beginning and end of the experiment, subjects indicated whether they felt *sad*, *angry*, *happy*, *surprised*, *fearful*, *disgusted,* or *relaxed*. The common six discrete emotions (Ekman et al. 1971) were supplemented by *relaxation*, which features in some theoretical models (Harmon-Jones et al. 2016) and may be a promising counterpart to VIMS.

#### Galvanic skin response

We recorded the galvanic skin response (GSR) to assess arousal and complement the SAM with an objective measure. While an increase in GSR cannot be attributed to a specific emotion, it does provide evidence of a change in arousal state. The GSR was collected for a total of 30 min and started after the subjects had completed the pre-questionnaire. We used the average number of peaks per minute to evaluate the data and controlled the accuracy of the values by analyzing the recorded noise signals. To measure the GSR, we used the Sensor from Shimmer3 GSR + Unit. The wireless sensor weighs 28 g and has a MSP430 microcontroller (24 MHz, MSP430 CPU), an 8 GB micro SD card, and a 450mAh rechargeable Li-ion battery. The measurement range of the device is 10 k-4.7MΩ (0.2uS–100uS) ± 10%. 22 k-680kΩ (1.5–45uS) ± 3%, detecting frequencies from DC to 15.9 Hz (Shimmer (n.d.)). For real-time recording of GSR, we instructed subjects to position the sensor strap above the wrist. Then, one electrode of the sensor was attached to the index finger and another to the middle finger.

#### Other measures

At the end, we asked how well the subjects had liked the stimuli. The rating ranged from *not at all liked* (1) to *very much liked* (7). They also rated how strongly they perceived their presence in the computerized world from *not at all* (0) to *very strongly* (100). Besides these measures, we tracked head movements and performance data during the starfield simulation (see “Simulation and time-to-contact task”). Finally, the participants’ eating and sleeping behavior as well as the *Positive and Negative Affect Schedule* (Breyer and Bluemke 2016) were recorded for other research projects.

### Subjects

Exclusion criteria were known health problems such as damage to the vestibular organs, or eye diseases that restrict vision and cannot be corrected by wearing glasses or contact lenses. For ethical reasons, subjects were offered to stop the experiment early as soon as they reported an FMS score of 15 or higher.

The required sample size of *n* = 66 (with alpha = 0.05, power = 0.80) was determined using an a priori power analysis with G-Power version 3.1.9.2 (Faul et al. 2009). The effect size (*f* = 0.26) was calculated following the study by Keshavarz and Hecht (2014), in which the interaction of time and music for FMS nausea scores yielded *η*_*p*_^2^ = 0.06. A total of 74 subjects were recruited, anticipating some dropouts and premature terminations. For recruitment, an advertisement was prepared that included initial information about the experiment, exclusion criteria, and a link to schedule an appointment. The subjects were assigned to one of the two groups (positive or neutral).

After collecting all data, we excluded two subjects from the analysis because of extremely high pre-SSQ total scores, which could be identified as outliers in an SSPS boxplot analysis (beyond 1.5 interquartile ranges from the median, with pre-SSQ total score of 67.46 or higher). Due to severe nausea/dizziness or severe discomfort, four additional subjects, including three from the neutral group and one from the positive group, chose to terminate the experiment within the first 5 min. In addition, there were 15 other subjects who terminated the experiment at a later stage. Since enough data could be recorded in these cases, the subjects were not excluded from the data analysis. After dropouts, 68 participants remained in our sample for data analysis. Hence, with the calculated sample size being *n* = 66, sufficient power of the data analysis was ensured.

### Materials and apparatus

We immersed the subjects in the virtual environment through an HTC Vive Cosmos HMD. While the subjects sat on a stationary chair with armrests, they held the associated HTC Vive Cosmos controller in their dominant hand, which was used to collect performance data. The Vive Cosmos features one LCD per eye. Each has a refresh rate of 90 Hz and a resolution of 1440 × 1700, resulting in a field of view of 97° horizontal and 94° vertical (Brown; HTC Corporation, n.d.). It weighs 702 g and features integrated stereo speaker. The Vive Cosmos has a hardware-adjustable eye comfort setting that allows subjects to manually adjust the interpupillary distance (IPD) between 61 and 72 mm.

The Vive Cosmos uses inside-out tracking where the position and orientation of the HMD in the environment are determined by cameras attached to the HMD. To track the HMD position, the Vive Cosmos has a built-in accelerometer and gyroscope, as well as six onboard cameras. The Cosmos controllers also have a built-in accelerometer and gyroscope. The HMD tracks the position and orientation of the controllers via a distinctive light pattern on a ring integrated into the controller design (Robertson 2019). The visor of the HMD can be flipped up and is thus more suitable for people who wear prescription glasses.

For the experiment, we connected the Vive Cosmos to an Alienware Area-51M R2 laptop with an Intel^®^ Core™ i9-10900K CPU (3.70 GHz) processor, 64 GB (DDR4) RAM, and a GeForce RTX 2080 SUPER™ (8 GB GDDR6). The VR application ran on a Windows 10 Enterprise operating system (version 20H2).

### Simulation and time-to-contact task

The VR application for the experiment was created with Unity 2019.4.29f1 and included an emotion induction for one minute and a starfield simulation for 14 min. Throughout the simulation, subjects sat on a comfortable chair.

First, subjects saw ten IAPS images for emotion induction, each image shown for 6 s. The images were either neutral or positive depending on the assigned group of the subject. The order of the images was the same for all subjects of a group. The image was shown in the middle of the HMD’s field of view, of which it occluded approximately 70%.

Second, after viewing the single IAPS images, we immersed subjects in a simulation of a white starfield moving around them. The starfield simulation was adapted from previous work on VIMS by Hemmerich et al. (2020) and included rotations. The parameters for the starfield simulation can be found in the supplementary materials. The random starfield movement around the subjects was simulated using the Jitter plug-in version 1.3 for Unity (Virtual Escapes 2019). To construct comparable motion for all subjects, a random seed initialized motion profiles with respect to all three axes (*x*, *y*, and *z*). The motion varied in amplitude, magnitude, and frequency to induce VIMS.

**Fig. 1:**
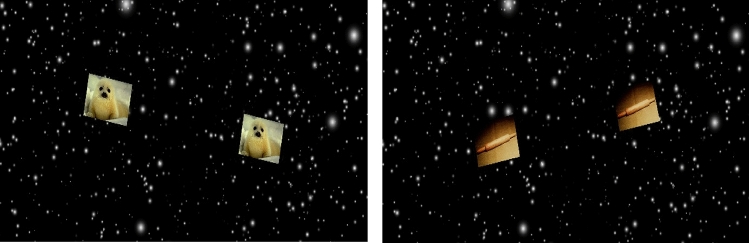
Virtual environment during the experiment. The environment consisted of blurred white spots (stars) and two positive (left) or neutral images (right)

While being immersed in the starfield simulation, subjects completed a time-to-contact (TTC) estimation task, which was used to induce emotions, focus attention on the visual motion, and assess performance. Within the starfield, two equal IAPS images moved toward each other and then disappeared. We instructed subjects to press the R2 button on the Vive controller immediately when the centers of the objects would have collided if they had continued their trajectory. Based on this input, TTC error was calculated (constant and absolute error). The images appeared 7 m apart from each other (center to center). They were 1.6 × 1.2 m in size and 8 m in front of the participants. The trajectory between the centers of the images was coupled to the translation and rotation of the starfield, but randomly rotated around the y-axis by either 25, −25 or 0 degrees. The images were displayed for three seconds before they moved toward each other. Random samples generated similar but not equal trajectories while the target images approached each other. The images disappeared shortly before their centers collided, which was between 0.6 and 1.5 s after they had started to move (see Fig. 1). The task then continued for an additional seven minutes, resulting in 14 min for the starfield simulation, not including the time taken by the subject to complete the SAM scale. Overall, subjects completed 120 trials for the whole simulation.

### Selected stimuli for inducing emotions

In our experiment, emotions were induced twice. The positive and neutral stimuli were selected based on detailed fundamental research and pre-tested in a pilot study. According to Joseph et al. (2020), video sequences with instruction are considered the most effective method for inducing positive emotions. However, since the videos recommended in the literature were not available or not suitable for the purposes of the present study, we tested videos it in a pilot study. The 15-min pilot study was conducted via the online survey tool *Unipark* and completed by a total of ninety subjects (*M* = 32.53 years, SD = 11.01). During the study, we compared four positive and two neutral videos. At the beginning, subjects were asked to provide some demographic information (*gender, age, education*), as well as to complete the SAM and a questionnaire about discrete emotions (see Emotion measures). One of the six videos was then played, randomized by individual gender to exclude gender-specific perceptual bias. After watching the video, subjects were again asked to complete the SAM and a questionnaire about discrete emotions, as well as to rate the video they had seen. Using mean comparisons of the SAM scales of valence and arousal, we identified one positive *Happy Moments* video (SAM scale valence (*M* = 6.53, SD = 1.51), SAM scale arousal (*M* = 5.27, SD = 1.53)) as the best stimulus for inducing positive emotions and the neutral *Berlin Traffic Camera* video (SAM scale valence (*M* = 5.53, SD = 1.64), SAM scale arousal (*M* = 5.07, SD = 2.34)) as the stimulus for inducing neutral emotions. In the *Happy Moments* video, various clips of happiness showed laughing children or babies, happy family celebrations, or emotional everyday moments. The *Berlin Traffic Camera* video showed a montage of different live cameras in Berlin, which recorded traffic in the city. We presented the videos on a 55-inch TV at a distance of 2.5 m from the subject, who was instructed to focus on the video content and to accept the upcoming emotions. For comparability of the groups, we shortened the videos to the same length (2 min 35 s).

For the second manipulation, we used selected images of the *International Affective Picture System* (IAPS) (Lang et al. 1997) and presented them to our subjects within the simulation (see “Simulation and time-to-contact task”). The IAPS is a widely used database in research that contains a large set of standardized, emotionally appealing, international color photographs that represent content from a wide range of semantic categories. In this process, the images had been rated using SAM scales and expanded into a set of normative emotional stimuli for the experimental study of emotion (Lang et al. 1997). In selecting the IAPS images, we excluded all images that did not elicit positive or neutral emotions, were of insufficient quality, or did not fit the purpose of the study (e.g., images of food). The final selection of IAPS images was made by considering the scores of the SAM scales valence and arousal. Accordingly, the positive group was presented with IAPS images that scored above 7.4 on the SAM valence scale and above 4 on the SAM arousal scale and the neutral group was shown IAPS images that scored between 4 and 5.6 on the SAM valence scale and below 3 on the SAM Arousal scale.

### Procedure

**Fig. 2:**
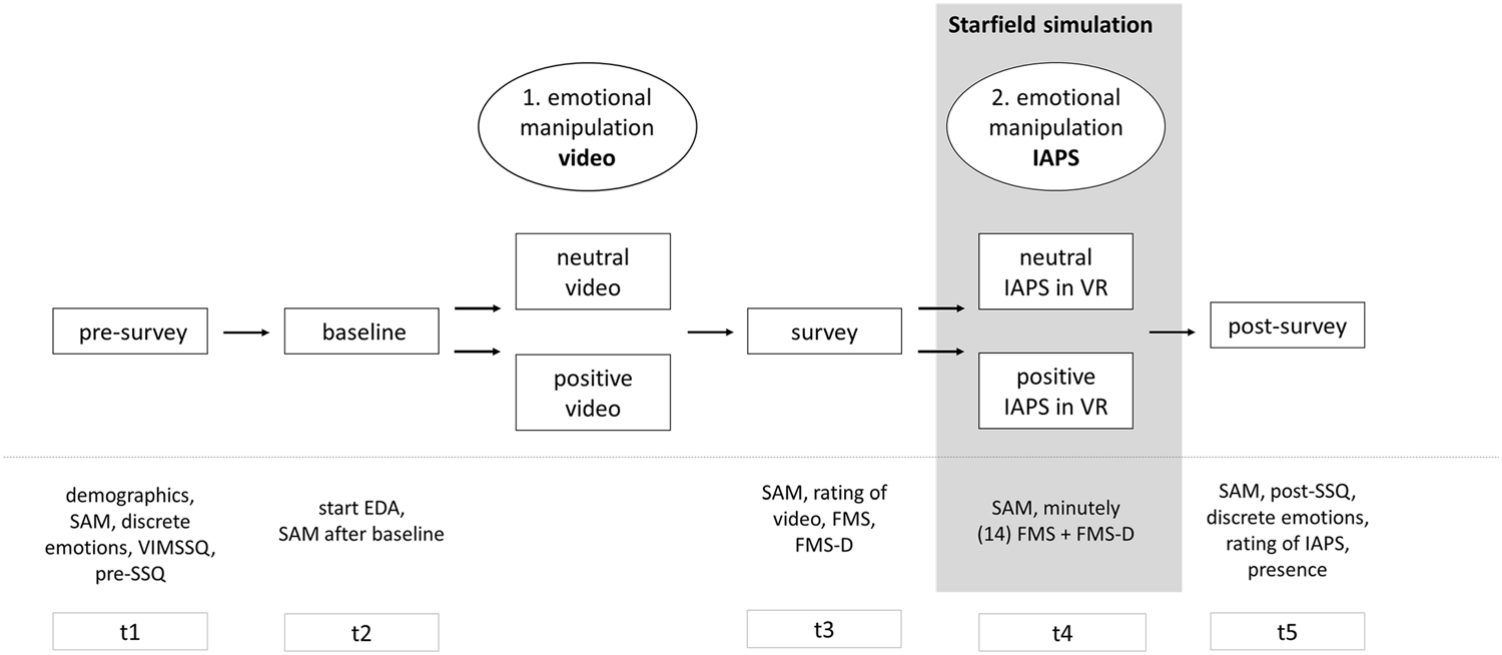
Experimental procedure. *SAM* Self-Assessment Manikin, *SSQ* Simulator Sickness Questionnaire, *FMS* Fast Motion Sickness Scale, *FMS-D* fast motion sickness for dizziness, *VIMSSQ* Visually Induced Motion Sickness Susceptibility Questionnaire, *GSR* galvanic skin response, *t1* initial state, *t2* after baseline, *t3* after video, *t4* in simulation, *t5* after simulation

Our experiment contained five measurement time points (t1–t5) and lasted approximately 1 h. The procedure is illustrated in Fig. 2. At the beginning, subjects were asked to sign a written informed consent form and to complete a demographic questionnaire, the pre-SSQ, VIMSSQ, SAM, and the questionnaire about discrete emotions (t1).

Afterward, we started the measurement of the GSR and collected baseline data to capture the initial status. For the baseline measurement, we asked subjects to relax, breathe regularly, and focus on the black screen, which showed a white crosshair. During video presentation (first emotion induction), the room was darkened. After the 5-min baseline measurement, we queried the subjects' emotional state using the SAM (t2).

After the first emotion induction, we presented the SAM, an item to evaluate the video, and a detailed explanation of the two FMS scales (t3). Subsequently, we explained the tasks to be performed during the VR exposure and helped subjects to put on the VR HMD. During this process, we ensured that the subjects were able to see the simulation in focus and IPD was properly adjusted.

During the 14-min exposure to the starfield simulation, the second emotion induction occurred using the selected IAPS images and the TTC task was completed. The subjects were not given specific instructions about their head movements and were asked to verbally rate their sickness every minute, using the FMS and FMS-D. The simulation was terminated whenever subjects wanted to stop due to severe VIMS or as soon as the simulation reached the end. Halfway through, the simulation and TTC task were paused, and the SAM scale was displayed (t4).

The subjects then completed the post-SSQ, the SAM, the questionnaire about discrete emotions, an evaluation of IAPS images, and the question about experienced presence (t5). Finally, they were debriefed and left the laboratory once VIMS-related symptoms had subsided. Due to the ongoing COVID-19 pandemic, a hygiene concept was developed that included the usual hygiene precautions, such as hand disinfection and wearing a face mask before and after, but not during the experiment.

## Results

All analyses were performed using Statistical Package for Social Sciences (SPSS) version 25.0 or R version 4.1.2. The a priori significance level was set to *p* < 0.05. For correlation analysis, we used the Spearman-Brown formula, since the collected data were not normally distributed (Shapiro-Wilk < 0.05). Since both ANOVAs (see Blanca et al. 2017) and MANOVAs (see Yatim and Ismail 2002) have been shown to be relatively robust to violations of the normality assumption, we preferred to perform parametric tests.

### Sample characteristics and baseline differences

A total of 68 subjects (30 female, 37 male, 1 diverse) with a mean age of 26.63 years (SD = 5.73) were included in the analysis. Our sample was recruited among students and research staff, all of whom had a high school diploma and most possessed a bachelor’s degree. The distribution of subjects between the positive (*n* = 33) and neutral (*n* = 35) groups, as well as the mean values of age, gender, and VIMSSQ score are presented in Table 1. The sample had very little VR experience, only six subjects reported one-time monthly use and one subject weekly use of VR.

**Table 1:** Sample description by groups for age, gender, and visually induced motion sickness susceptibility questionnaire

	Neutral	Positive	Total
*N*	35	33	68
Age	25.74 (3.96)	27.55 (7.10)	26.63 (5.73)
Gender	17 *f*, 18 *m*, 0 *d*	13 *f*, 19 *m*, 1 *d*	30 *f*, 37 *m*, 1 *d*
VIMSSQ	6.23 (3.73)	5.45 (2.84)	5.85 (3.33)

For age and VIMSSQ, values indicate *M* (*SD*); *VIMSSQ* Visually Induced Motion Sickness Susceptibility Questionnaire, *f* female, *m*  male, *d*  diverse

We found no significant differences between groups in age (*t*(66) = 1.30, *p* = 0.197), gender (*χ*^2^(2) = 1.50, *p* = 0.472), SAM scale valence t1 (*t*(66) = 1.26, *p* = 0.211) and t2 (*t*(66) = 0.45, *p* = 0.657), and VIMSSQ score (*t*(66) =  −0.96, *p* = 0.342). Furthermore, we included a *t*-test for independent samples on the pre-SSQ scores with *group* as between-subjects factor. Using the subscale scores nausea (*t*(66) = −0.22, *p* = 0.828), oculomotor (*t*(66) = −0.72, *p* = 0.475), disorientation (*t*(66) = −0.46, *p* = 0.648), and the SSQ total score prior to VR exposure (*t*(66) = −0.58, *p* = 0.565), we found no significant group differences (see Table 2 for SSQ and FMS mean values). This suggests that the groups did not differ in terms of VIMS-related symptoms before exposure.

**Table 2:** Mean (SD) pre-SSQ and post-SSQ scores and mean (SD) peak FMS and FMS-D scores

VIMS measures	Group		Total
	Positive	Neutral	
pre-SSQ			
Nausea	14.45 (14.92)	15.26 (15.56)	14.87 (15.14)
Oculomotor	21.59 (15.64)	24.47 (17.35)	23.07 (16.48)
Disorientation	8.86 (11.45)	10.34 (14.85)	9.62 (13.23)
Total	18.70 (13.65)	20.84 (16.61)	19.80 (15.17)
post-SSQ			
Nausea	65.33 (49.29)	80.14 (57.48)	72.95 (53.78)
Oculomotor	42.95 (29.82)	54.14 (34.89)	48.71 (32.78)
Disorientation	75.51 (62.06)	86.30 (70.96)	81.06 (66.52)
Total	67.09 (46.24)	81.32 (54.41)	74.42 (50.74)
FMS peaks	7.76 (6.71)	9.09 (6.18)	8.44 (6.43)
FMS-D peaks	9.61 (5.83)	11.31 (5.49)	10.49 (5.68)

*SSQ* Simulator Sickness Questionnaire,* FMS* Fast Motion Sickness Scale, *FMS-D* fast motion sickness for dizziness

### Manipulation check

For all 68 subjects, performance data (head movement and TTC task data) could be recorded during the VR exposure. On average, subjects completed 86.67% of the 120 tasks presented to them (*M* = 104 collisions, SD = 26.18) and skipped 2.5% (*M* = 3 collisions, SD = 3.63). Due to premature dropouts, VR exposure was completed on average after 89.17% collisions (*M* = 107, SD = 26.52). Given the small number of missed tasks, we assume that the task was performed as instructed and that subjects were exposed to the VIMS-inducing simulation. The average of the TTC constant error was 0.22 s (SD = 0.31).

**Fig. 3:**
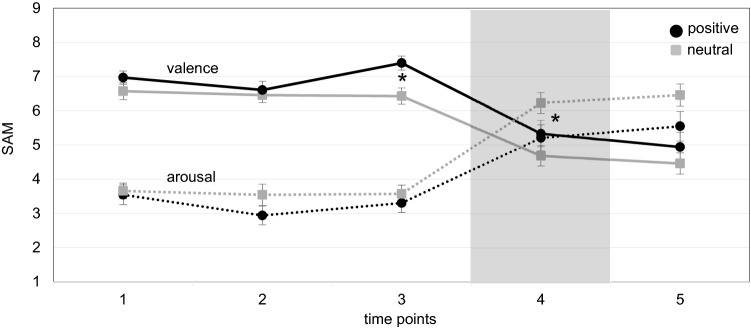
Mean values of the SAM scales valence and arousal divided by groups. *SAM* Self-Assessment Manikin, Error bars represent standard errors of the mean; *1* initial state, *2* after baseline, *3* after video, *4* in simulation, *5* after simulation; Gray shaded area indicates the period of VR exposure within the experiment

To check for successful emotion induction, we compared the means of the SAM scales valence and arousal using a mixed 5 × 2 MANOVA with the within-subjects factor *time* (t1–t5) and the between-subjects factor *group* (positive and neutral) (see Fig. 3). The MANOVA yielded a significant main effect of time, *F*(8, 528) = 28.78, *p* < 0.001, *η*_*p*_^2^ = 0.30. Furthermore, we could not find an effect of *group*, *F*(2, 65) = 1.87, *p* = 0.162, or an interaction effect of *time* and *group*, *F*(8, 528) = 1.78, *p* = 0.077.

To control whether the emotion induction had been successful at the measurement time points after emotion induction (t3 and t4) in more detail, we conducted post hoc *t*-tests for independent samples. Despite nonsignificant main and interaction effects, we undertook this analysis to gain further understanding of the data, in particular whether any of the emotion inductions elicited differences in valence and how long they lasted. The positive emotion induction did indeed produce higher valence ratings (SAM) than the neutral group at measurement time point t3, *t*(66) = 3.08, *p* = 0.003, *d* = 0.74, whereas the groups did not differ in arousal, *t*(66) = −0.71, *p* = 0.479. During the task, at measurement time point t4, the SAM scale valence difference had disappeared, *t*(66) = 1.34, *p* = 0.184, but SAM scale arousal ratings were now higher for the neutral group, *t*(66) = −2.11, *p* = 0.038, *d* = 0.51. Beyond that, we found no significant differences between the groups. Our results show that emotion induction was successful but note that the VIMS-provoking simulation changed the emotional state of the subject quite succinctly. With immersion, arousal went up and valence went down. That is, subjects did focus on the task and thus on the nauseating stimuli and, as a result, their emotional valence was reduced.

We also asked how well subjects liked the stimuli presented to them. Using *t*-tests for independent samples, we found significant mean differences for both the video (positive (*M* = 5.58, SD = 1.03), neutral (*M* = 3.54, SD = 1.36), *t*(66) = 6.92, *p* < 0.001, *d* = 1.69) and for the IAPS images (positive (*M* = 4.58, SD = 1.25), neutral (*M* = 3.49, SD = 1.36), *t*(66) = 3.44, *p* < 0.001, *d* = 0.84).

### Influence of induced emotions

A mixed 2 × 2 ANOVA on the SSQ scores with within-subjects factor *time* (pre-post) and between-subjects factor *group* revealed significant main effects of *time* (pre–post) for the total score (*F*(1, 66) = 89.30, *p* < 0.001, *η*_*p*_^2^ = 0.58) and all subscales (nausea: *F*(1, 66) = 86. 45, *p* < 0.001, *η*_*p*_^2^ = 0.57, oculomotor: *F*(1, 66) = 49.35, *p* < 0.001, *η*_*p*_^2^ = 0.43, disorientation: *F*(1, 66) = 86.63, *p* < 0.001, *η*_*p*_^2^ = 0.57). This indicates that SSQ scores were higher after VR exposure than before (see Table 2). Additionally, we could not find a main effect of *group* regarding to SSQ total scores (*F*(1, 66) = 1.36, *p* = 0.247) or for the subscales nausea (*F*(1, 66) = 1.15, *p* = 0.287), oculomotor (*F*(1, 66) = 1.90, *p* = 0.172), and disorientation (*F*(1, 66) = 0.48, *p* = 0.489). Furthermore, *time* and *group* did not interact for the SSQ total scores (*F*(1, 66) = 1.10, *p* = 0.298) or the subscales nausea (*F*(1, 66) = 1.26, *p* = 0.265), oculomotor (*F*(1, 66) = 1.31, *p* = 0.257), and disorientation (*F*(1, 66) = 0.37, *p* = 0.545).

To further investigate the influence of induced emotions, we performed analyses with FMS and FMS-D. A between-subjects one-factor MANOVA for the FMS and FMS-D mean peak scores yielded no main effect of *group* (*F*(2, 65) = 0.80, *p* = 0.453). In addition, we consulted the FMS and FMS-D time course for analysis. A mixed 15 × 2 MANOVA with the within-subjects factor *time course* (15 measurement time points) and the between-subjects factor *group* revealed a significant main effect of *time course* (*F*(28, 1848) = 28.05, *p* < 0.001, *η*_*p*_^2^ = 0.30), indicating increasing FMS and FMS-D scores over time of VR exposure. Furthermore, we did not detect a significant main effect of *group* (*F*(2, 65) = 0.97, *p* = 0.386) or an interaction of *time course* and *group* (*F*(28, 1848) = 0.36, *p* = 0.999). Thus, inducing positive emotions with video and pictures was not able to lower the experienced VIMS. However, it should be noted that the FMS values of the positive group are descriptively slightly lower during the time course (see supplementary materials).

### Correlations between emotions and VIMS

Emotional valence and VIMS were nonetheless strongly related. Table 3 shows that the SAM scale valence correlates significantly negatively with the FMS peaks and FMS-D peaks at all five measurement time points. The correlations at measurement time points t1–t3 are significant but moderate according to the classification by Cohen (1988). At measurement time points t4 and t5, correlations were strong. The results suggest that the initial emotional state has an influence on the FMS scores. During exposure, valence becomes more negative due to the negative symptoms (see also Fig. 3).

**Table 3:** Correlations between SAM scales valence and arousal and VIMS measures

VIMS measures	Valence t1	Valence t2	Valence t3	Valence t4	Valence t5
post-SSQ total	−0.08	−0.18	−0.18	−0.67**	−0.68**
FMS Peaks	−0.27*	−0.33**	−0.30**	−0.75**	−0.71**
FMS-D Peaks	−0.25*	−0.30**	−0.30**	−0.71**	−0.64**

VIMS measures	Arousal t1	Arousal t2	Arousal t3	Arousal t4	Arousal t5
post-SSQ total	0.31**	0.23*	0.32**	0.66**	0.66**
FMS Peaks	0.21*	0.22*	0.32**	0.69**	0.65**
FMS-D Peaks	0.29**	0.29**	0.30**	0.65**	0.68**

*p < 0.05, **p < 0.01, one-tailed Spearman rank correlations; *SSQ* Simulator Sickness Questionnaire, *FMS* Fast Motion Sickness Scale; *FMS-D* Fast Motion Sickness Scale for Dizziness, *t1* initial state, *t2* after baseline, *t3* after video, *t4* in simulation, *t5* after simulation

Furthermore, the SAM scale arousal correlates significantly positive with all VIMS questionnaires at all five measurement time points (see Table 3). At measurement time points t1–t3 the correlations between arousal and FMS and FMS-D are moderate and high at measurement time points t4 and t5. During the experiment, we recorded the phasic GSR data of 65 subjects based on the peaks per minute at measurement time points baseline (t2), video (t3), and VR (t4). Due to technical recording error, one complete data set and two measurements at t4 for the GSR are missing. The GSR peaks per minute at t4 are significantly positively correlated with SAM scale arousal at the measurement time points t1 (*r*_*s*_ = 0.25), t4 (*r*_*s*_ = 0.32) and t5 (*r*_*s*_ = 0.22). Finally, we found significant positive correlations between GSR peaks per minute at measurement time point t4 (*M* = 6.44, SD = 2.89) and post-SSQ total score (*r*_*s*_ = 0.26) as well as FMS peaks (*r*_*s*_ = 0.39) and FMS-D peaks (*r*_*s*_ = 0.26). We were unable to document a relationship between the VIMS measurements and the GSR peaks per minute at the baseline (*M* = 2.55, SD = 2.64) and video (*M* = 4.12, SD = 3.36) measurement time points. Overall, our results indicate that the initial level of self-reported arousal might influence the subsequent symptom development. During simulation, the arousal increased due to adverse symptoms (see also Fig. 3).

For exploratory reasons, we queried discrete emotions at two different measurement time points (t1 and t5) and correlated them with the VIMS questionnaires. At measurement time point t1 sadness and FMS-D peaks were significantly positively correlated (*r*_*s*_ = 0.30) with each other. Beyond that, we did not find any significant correlations between the discrete emotions at measurement time point t1 and the VIMS measures. Furthermore, we were able to detect significant positive correlations between the discrete emotions sadness, anger, surprise, disgust, and fear at measurement time point t5 and all VIMS questionnaires (see Table 4). Additionally, the discrete emotions happiness and relaxation correlated negatively with VIMS at measurement time point t5. The negative correlation between relaxation and peak FMS (*r*_*s*_ = −0.69) was the highest. Furthermore, relaxation and arousal measured by the SAM appear to represent overlapping concepts. As relaxation collected at t5 correlates significantly negatively with arousal at t1 (*r*_*s*_ = −0.24), t2 (*r*_*s*_ = −0.27), t3 (*r*_*s*_ = −0.36), t4 (*r*_*s*_ = −0.53), and t5 (*r*_*s*_ = −0.63).

**Table 4:** Correlations between discrete emotions at t5 and VIMS measures

Discrete emotions	*M* (SD)	post-SSQ total	Peak FMS	Peak FMS-D
Sadness	1.22 (0.45)	0.34**	0.36**	0.33**
Anger	1.15 (0.53)	0.28*	0.29*	0.26*
happiness	2.62 (0.90)	−0.44**	−0.49**	−0.38**
surprise	2.54 (1.29)	0.43**	0.41**	0.33**
Fear	1.26 (0.59)	0.39**	0.35**	0.41**
Disgust	1.38 (0.88)	0.48**	0.49**	0.37**
relaxation	2.26 (1.09)	−0.65**	−0.69**	−0.60**

*p < 0.05, **p < 0.01; two-tailed spearman rank correlations; *SSQ* Simulator Sickness Questionnaire, *FMS* Fast Motion Sickness scale, *FMS-D* Fast Motion Sickness Scale for Dizziness

### Other results

Non-parametric Spearman rank correlations between presence, head movement, TTC task errors and VIMS symptoms showed a significant positive correlation between presence and VIMS (see Table 5). In addition, we found a correlation between head movement and FMS peaks as well as FMS-D peaks. The constant error in the TTC task and the FMS-D were significantly correlated.

**Table 5:** Correlations between presence, head movements, TTC task errors and VIMS measures

Variables	*M* (SD)	1	2	3	4
1. Presence	52.07 (27.98)				
2. Head movement	0.33 (0.38)	0.17			
3. TTC constant error	0.22 (0.32)	0.18	0.15		
4. TTC absolute error	0.05 (0.34)	−0.02	−0.02	0.05	
5. post-SSQ total	74.42 (50.74)	0.24*	0.18	0.11	−0.05
6. FMS peaks	8.44 (6.43)	0.22*	0.23*	0.13	−0.11
7. FMS-D peaks	10.49 (5.68)	0.18	0.22*	0.28**	−0.02

*p < 0.05, **p < 0.01, one-tailed Spearman rank correlations, *SSQ* Simulator Sickness Questionnaire, *FMS* Fast Motion Sickness Scale, *FMS-D* Fast Motion Sickness Scale for Dizziness, *TTC* time-to-contact

## Discussion

The aim of the present study was to investigate the influence of positive emotions on VIMS. For this purpose, we directly induced positive or neutral emotions by using videos before the provocative VR simulation and embedded positively or neutrally valenced images during the simulation. The results of the manipulation check showed that the videos played before the simulation successfully induced the desired emotions. However, they did not transport to the provocative task. There, we could not find differences in subjects' valence during the simulation corresponding to the neutral or positive emotional manipulation. We found no differences in terms of VIMS symptomology between the neutral and positive emotion group. However, we did find significant associations between emotions and VIMS independently of the manipulation. Subjects who initially experienced more positive emotions perceived fewer VIMS symptoms during and after simulation. Conversely, subjects with a negative emotional state prior to the simulation developed more severe VIMS symptoms. Not surprisingly, subjects with higher VIMS symptomatology in the VR exposure experienced fewer positive emotions at that time. Similarly, higher levels of arousal at baseline as measured by self-report were associated with more severe VIMS symptoms. Furthermore, we found moderate to strong correlations between VIMS and discrete emotions collected after the simulation. Thus, emotions and VIMS are correlated but not easily manipulated.

### Influence of induced emotions on VIMS

Contrary to our assumption, we found no differences between the neutral and positive emotion groups in terms of VIMS symptom level. These findings are inconsistent with the notion that the reported VIMS-reducing effects of pleasant stimuli, such as pleasant music, odor, and taste(Kaufeld et al. 2022a; Keshavarz et al. 2015; Keshavarz and Hecht 2014; Peck et al. 2020), are transported by emotion. Note, however, that subjects in the aforementioned studies experienced rather mild to moderate VIMS. It is thus also possible that our first successful emotion induction before the simulation (videos) was overshadowed by the comparatively severe symptoms that occurred when the provocative simulation began. Similarly, the second emotion induction (IAPS images embedded in the simulation) in our study, might not have been effective enough to alleviate severe symptoms. For instance, in (Keshavarz et al.'s 2015) study, the rose odor had a mitigating effect only when it was consciously perceived. Furthermore, in contrast to the cited previous studies, we chose pleasant visual stimuli that had found to be most promising for emotion induction in the literature (Joseph et al. 2020; Siedlecka and Denson 2019). However, this may not have been the best option for emotion induction in the present case. Although visual stimuli are superior to other modalities to induce an emotional state, it may have been suboptimal for the VR exposure, which also used the visual channel. In sum, the VIMS symptoms we induced have likely been too strong to find evidence for subtle alleviating effects of positive emotion induction.

### The relation of emotions and VIMS

Nonetheless, we found that emotions do play a role in the development of VIMS. Pre-simulation valence was moderately negatively related with VIMS symptoms during the simulation, in the sense that positive emotional predisposition may not be easily induced, but does protect against the development of VIMS symptoms. Prior negative valence may favor the development of symptoms. This is consistent with studies showing that baseline emotional state has an impact on coping with disease symptoms (Ashkhaneh et al. 2015; Cohen and Pressman 2006; Fredrickson 1998; Gil et al. 2004; Olver et al. 2014). Moreover, we showed that the correlation between valence and VIMS strengthened at the measurement time points within and after the simulation. We assume that in this case the unpleasant symptoms caused by the simulation directly and negatively influenced the mood of the subjects. Similarly, Van der Spek et al. (2007) found that individuals with more severe VIMS symptoms rated virtual environments as less pleasant. Reuten et al. (2021) likewise found an association of VIMS and unpleasantness during a simulation.

We also found relationships between VIMS and self-reported arousal. We were able to reveal a moderate positive correlation of VIMS with arousal captured before the simulation as well as a strong positive correlation with arousal captured during and after the simulation. This appears to indicate that initial arousal has a small influence upon VIMS, but more so does VIMS seem to strongly increase arousal. This agrees with the considerations in Mittelstaedt’s (2020) review that higher sympathetic tone goes along with VIMS susceptibility. This is consistent with the modest correlation of self-reported arousal, but not with the absence of a correlation with pre-exposure GSR. For the measurement time points during and after the simulation, in contrast, we found significant correlations between VIMS symptoms and arousal for both self-reports as well as GSR, which is in line with other studies showing VIMS measurement by GSR (Gavgani et al. 2017; Kim et al. 2005; Wan et al. 2003).

The analysis of discrete emotions before the experiment showed no significant results apart from the association of sadness and disorientation (FMS-D), suggesting that it is exceedingly hard to investigate how discrete emotions may influence VIMS. Analysis of the discrete emotions anger, sadness, surprise, disgust, and fear after the simulation showed positive relations, whereas analysis of the emotions happiness and relaxation showed negative relations with VIMS. The link between low VIMS and relaxation was particularly high. The character of the analysis on discrete emotions is very exploratory, showing mainly what kind of emotions are elicited or not elicited when VIMS is experimentally induced. Moreover, the strong negative relationship of VIMS and relaxation suggests that relaxation is an antagonist of VIMS, which is consistent with our findings that subjects with low arousal and high valence reported low VIMS symptoms. Similarly, Keshavarz and Hecht (2014) found a negative trend between relaxing music and VIMS. Relaxation and arousal appear to be complementary concepts, as arousal is correlated positively at the same rate with VIMS as relaxation is correlated negatively with VIMS. This points to the potential benefit of relaxation instructions to alleviate VIMS symptoms. An instruction to relax may be more meaningful than an instruction to be less aroused.

Within the framework of sensory conflict theory, we suggest that positive emotions may distract from the perception of sensory conflict and modulate the perception of aversive symptoms. Previous studies showed that emotions can influence body movement (e.g., Bonnet et al. 2008; Michalak et al. 2009). Since postural instability is often associated with VIMS (see Riccio and Stoffregen 1991), further explanations of the relationship between emotions and VIMS can be discussed in this framework. Exploratively, we found a small negative correlation (*r*_*s*_ = −0.25) between measured head movement and valence at t2 only, which indicates that lower valence is associated with slightly larger head movements. At the same time, lower valence at this stage is also related to more VIMS. Thus, the results are compatible with postural instability theory. Note, however, that this is an ex-post observation and research on emotion and gait would suggest an opposite effect of emotion (Michalak et al. 2009). Further research with a specific hypothesis as to why positive emotions might lead to less head/body movement could pursue this venue. As a note of caution, the postural movements of our subjects were mainly driven by the task to keep the moving targets in view. A task that is more suitable to let emotion exert its effects on head and body movement would be required.

### Limitations and future directions

In the present work, the role of positive emotions on VIMS was specifically investigated using two ways of emotion induction. The first emotion induction using pre-tested videos successfully induced positive emotions according to the manipulation check. In combination with the second emotion induction using embedded IAPS images in the VR simulation, it did not achieve the desired effect of significantly lowering VIMS. Are emotion inductions plainly of limited use in the face of a strong nauseating stimulus? Or may the presentation of the widely tested but aging IAPS images not have sufficed because of limits in quality and sharpness of the images? We consider the former to be more likely, as the images were sufficiently recognizable. And we had deliberately chosen visual emotion induction since previous studies indicate that it is the most successful way to induce emotions (Joseph et al. 2020; Siedlecka and Denson 2019). Thus, the positive effects obtained by previous studies, which have shown, for example, that music is able to reduce VIMS (Keshavarz and Hecht 2014; Peck et al. 2020), are either not entirely transported by emotion, or they only apply to moderate cases of VIMS. Future studies should investigate whether visual emotion induction could be an effective countermeasure in less provocative virtual environments, e.g., integrated into a gaming scenario. Since other studies found the successful decrease of VIMS especially in mild symptoms, it is reasonable to further optimize emotion induction and to include a less VIMS-inducing simulation. In our study, we focused on positive emotions to derive possible implications for future countermeasures. For future studies, it would be of interest to also investigate the role of negative emotions on VIMS.

Some results of our study, such as the correlation of baseline valence and VIMS symptoms, could only be shown by the FMS and not by the SSQ. In our opinion, this can be explained by the fact that symptoms measured by the SSQ can only be collected before and after the simulation. The FMS captures symptoms during the simulation and is more sensitive, as symptoms often subside quickly after simulation. Also, the well-anchored FMS can be treated as an interval scale, which cannot be said for the SSQ.

### Practical implications

VR HMDs are increasingly used in professional contexts such as training and therapy and offer new opportunities for cost-effective and safe applications. Advances in VR technology are leading to better graphical representation, yet this cannot overcome the limitations imposed by VIMS symptoms. In the present study, although no concrete countermeasures could be derived, a deeper understanding of the role of emotions could be gained.

Our results suggest that VIMS and emotions are mutually dependent. A positive mood prior to the simulation could be a protective factor against VIMS symptoms. Whereas the symptomatology during the simulation strongly affected the current mood. Thus, if someone is in a bad mood before starting a simulation, that person might be more susceptible to VIMS and the emotional state might further deteriorate. Different baseline emotional states can be explained by individual experiences and emotion regulation strategies. Accordingly, the use of VR or simulators in professional training should optimally be based on individual decisions. When arousal is high and valence is strongly negative, e.g., when tension is experienced, relaxation techniques, might help to adjust baseline mood and minimize susceptibility. For VIMS countermeasures, emotion inductions that address not only the visual channel may be more effective, as this channel is strongly engaged by the perception of the virtual environment. However, further research is needed to verify these considerations. The results suggest a strong influence of the VR task on the subject’s emotional state. Thus, emotion induction might be more useful during the recovery process than during exposure.

## Conclusion

In the present study, we induced positive emotions in two ways and queried valence, arousal, and VIMS symptoms before, during, and after a provocative VR simulation at five measurement time points. Contrary to our expectations, visually induced positive emotions did not reduce symptoms of VIMS during or after the simulation. Nevertheless, we were able to shed further light on the role of emotions in VIMS symptoms. According to our results, the baseline emotional state may have an impact on VIMS, and more importantly, the severity of VIMS symptoms is related to arousal and negative valence of emotion. The interdependence of emotions and VIMS should be considered when developing future countermeasures to make VR experiences more pleasant and to increase user acceptance. Further investigation is needed to determine the extent to which techniques that target an emotional state of positive valence and low arousal (e.g., relaxation techniques) might alleviate VIMS.

## Supplementary Information

Below is the link to the electronic supplementary material.

Supplementary file1 (PDF 153 KB)

## Declarations

### Data availability

The datasets generated during and/or analyzed during the current study are available from the corresponding author on reasonable request.
