# Supplementary material for: Emotions are associated with the genesis of visually induced motion sickness in virtual reality
Source: Exp Brain Res. 2022 Sep 6;240(10):2757–71. doi: 10.1007/s00221-022-06454-z (PMC9447355; doi:10.1007/s00221-022-06454-z)
Supplement: Supplementary file 1 — Supplementary file1 (PDF 153 KB) [file 221_2022_6454_MOESM1_ESM.pdf]

Supplementary materials

Table 1

Parameters for random translational and rotational motions. The values for the amplitude range are documented in meters for translational and in degrees for rotational motions.

Motion Axis	Seed	Magnitude	Amplitude Range	Frequency
Translation				
x	970822784	.75	(-1; 1)	2.11
y	970822784	.75	(-1; 1)	2.11
z	542916416	.75	(-1; 1)	.5
Rotation				
x	482038366	.75	(-25; 25)	2.05
y	115579920	1	(-25; 25)	1.59
z	542916416	1	(-90; 90)	1

Fig. 1

Time course of the FMS scores minute by minute separated by groups

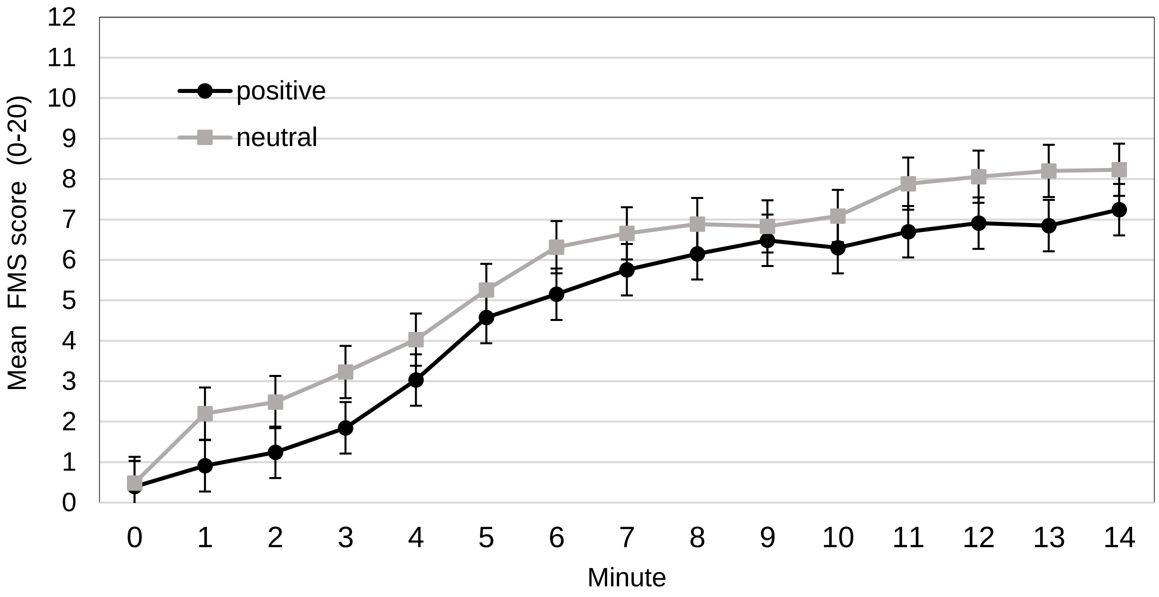

Note. Error bars represent standard error of the mean. FMS = Fast Motion Sickness Scale.

Fig. 2

Time course of the FMS-D scores minute by minute separated by groups

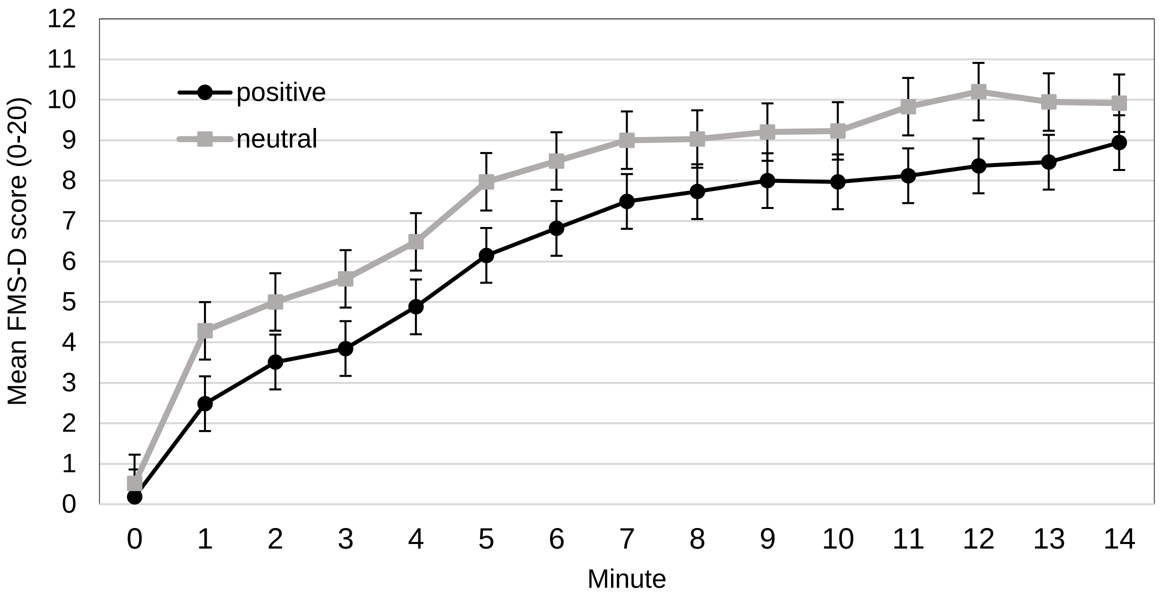

Note. Error bars represent standard error of the mean. FMS = Fast Motion Sickness Scale.
